# Reliability of automated brain volumetric analysis: A test by comparing NeuroQuant and volBrain software

**DOI:** 10.1002/brb3.3320

**Published:** 2023-11-23

**Authors:** Panagiotis Koussis, Panagiotis Toulas, Demetrios Glotsos, Eleni Lamprou, Demetrios Kehagias, Eleftherios Lavdas

**Affiliations:** ^1^ Bioiatriki SA/MRI Department Kifissias ave and Papada Athens Greece; ^2^ Department of Biomedical Sciences University of West Attika Athens Greece; ^3^ 1^st^ General High School of Psychiko Athens Greece

**Keywords:** atrophy, brain, comparison, NeuroQuant, volBrain, volumetry

## Abstract

**Background and purpose:**

Brain volume analysis from magnetic resonance imaging (MRI) is gaining an important role in neurological diagnosis. This study compares the volumes of brain segments measured by two automated brain analysis software, NeuroQuant (NQ), and volBrain (VB) in order to test their reliability in brain volumetry.

**Methods:**

Using NQ and VB software, the same brain segment volumes were calculated and compared, taken from 56 patients scanned under the same MRI sequence. These segments were intracranial cavity, putamen, thalamus, amygdala, whole brain, cerebellum, white matter, and hippocampus. The paired *t*‐test method has been used to determine if there was a significant difference in these measurements. The interclass correlation (ICC) is used to test inter‐method reliability between the two software. Finally, regression analysis was used to examine the possibility of linear correlation.

**Results:**

In all brain segments tested but hippocampus, significant differences were found. ICC presents satisfactory to excellent reliability in all brain segments except thalamus and amygdala for which reliability has been proven to be poor. In most cases, linear correlation was found.

**Conclusions:**

The significant differences found in the majority of the tested brain segments are raising questions about the reliability of automated brain analysis as a quantitative tool. Strong linear correlation of the volumetric measurements and good reliability indicates that, each software provides good qualitative information of brain structures size.

## 1 INTRODUCTION—THE ROLE OF AUTOMATED BRAIN VOLUMETRY

Automated brain volumetry plays a crucial role in clinical psychiatry and neurology, aiming to provide accuracy in a field where diagnosis often relies on subjective self‐reports and tests (Manjón & Coupé, 2016; Scarpazza et al., 2020).

Volume measurements of brain segments are strongly related to neurodegenerative diseases, such as Alzheimer's disease, frontotemporal dementia, multiple sclerosis, or epilepsy. Volumetric results have important applications in supporting disease diagnosis, monitoring treatment effect and help clinicians to improve their understanding on the disease mechanisms (Giorgio & De Stefano, 2013).

In the past, the segmentation and volume calculations of brain segments from magnetic resonance imaging (MRI) required manual procedures, which were time‐consuming and heavily depended on the expertise of investigators in neuroanatomical boundaries (Keller & Roberts, 2009). However, with the advent of automated brain volumetry analysis from MRI imaging, these limitations seem like to have been overcome. This modern computer‐aided diagnosis method offers, user‐friendly and fast results, targeting the improvement in efficiency and reliability of neuroimaging research and practice related to brain atrophy.

## 2 THE REASON FOR THIS RESEARCH

In our MRI laboratory (BIOIATRIKI), we had the opportunity to use NeuroQuant (NQ) as software for brain volume analysis, which was provided to us for limited time, as a donation by The Hellenic Academy of Neuro‐Immunology. Although we initially found the software satisfactory, we also explored alternative, more affordable options for the future and came across volBrain (VB) software, which is available under registration and free for 10 jobs per day. This prompted us to compare the results obtained from NQ with those from VB for the same sample of patients and brain structures. Any identified differences in volume measurements, particularly in certain brain structures, could raise doubts about the reliability of automated brain segment analysis and the interpretation of the results for atrophy determination.

## 3 METHODS AND MATERIALS

### 3.1 Patient handling

In this study, 56 adult patients (22 males/34 females), ages 18–70 years (with an average age 44 years), underwent MRI brain scans. These patients were being tested for multiple sclerosis (27/56, 48.2%) or dementia (21/56, 37.5%) based on the prescription of their clinical neurologist. We also had a small group of healthy volunteers (8/56, 14.3%). Patients with tumors or prior brain surgeries were excluded from the study, as these conditions could affect the measurements obtained from automated brain volumetry. We did not separate our data according to pathology type or from healthy individuals, as the study aimed to compare measurements from each software within the same individuals rather than defining measurements related to any specific pathology.

The research was conducted at BIOIATRIKI SA, MRI department, Athens, Greece, and was approved by the Scientific Council and the Bioethics Committee of BIOIATRIKI SA, Healthcare Provider Group, Athens, Greece, as well as the Bioethics Committee of the University of West Attica, Greece. All patients were fully informed about the study by reading an informative document and provided written consent to participate in it. Although they had the right to retreat from their decision, no‐one did. Their privacy and data protection were ensured according to the applicable General Data Protection Regulation protocols.

### 3.2 Scanning parameters

All scans were performed using General Electric's Healthcare, WI, USA, Discovery 3.0T MR system, using the same 3D T1 sequence without paramagnetic contrast enhancement. The sequence setting parameters were as follows: 3D/GRE/FSPGR sequence type, field‐of‐view = 25.6 cm, slice thickness = 1.2 mm, echo time = 2.4 ms, repetition time = 5.7 ms, inversion time = 600 ms, frequency/phase = 192/192, and band width = 31.25 kHz. The average acquisition time of the sequence was about 4 min. All images were anonymized immediately after each scan using MRI scanner's software (GE patient anonymized utility). Before any further processing, all scans were reviewed for their quality in relation to motion or other artifacts.

### 3.3 Volumetry software

NQ was developed by CorTechs Labs Inc. and has obtained FDA and CE approvals for clinical use. VB has been created by ITACA at the Valencia University, Spain in cooperation with the Pictura Research Group of the University of Bordeaux. VB is primarily intended for research purposes and does not possess any certifications by the time of the study.

The procedure was as follows: Access was available to “Multi Structure Report” and “General Report” from the reports’ options of the NQ internet platform. The initial DICOM images were zipped and transmitted to NQ. Subsequently, the same images were also analyzed by the VB software using “Volumetry Report” option from the VB internet platform (version 1.0 released 04/03/2015). The initial DICOM images were converted to NIFTI format, using ITK Snap software version 3.8.0, before they were transmitted to VB for processing. Each software has its own algorithms but there are some generally similar aspects in every procedure. The MRI scans are aligned or registered to a standard anatomical template to ensure consistent spatial reference across different subjects. This step involves mapping the individual brain structures to the template. The registered MRI scans are then segmented, which involves dividing the brain into different regions or structures of interest, this is typically achieved using intensity‐based classification but NQ and VB use atlas‐based segmentation methods. NQ pipeline inflates the brain to a spherical shape; maps the spherical‐shaped brain to a spherical space shared with the Talairach atlas coordinates; identifies all the segmented brain regions and deflates the brain to its original shape (Chung et al., 2020). In the VB pipeline, all segmentations process, except of volumes of white matter, gray matter, and cerebrospinal fluid, are based on different adaptations of a multi‐atlas patch‐based label fusion segmentation, on a library of manually labeled cases. The segmented brain regions are quantified by measuring their volume or size. This is done by counting the number of voxels (3D pixels) within each region or by estimating the volume based on the shape and dimensions of the segmented structures.

Each software produces a report to present the measurements of brain segments. The quantified volumes of the segmented brain regions are presented in a format that includes an exact volume measurement and a relative volume as a percentage of intracranial cavity volume (ICV). These values are compared to normative databases or age‐matched control groups. This allows the identification of potential deviations or abnormalities in the brain structures, such as atrophy or enlargement. These reports can be used by healthcare professionals to assist them in the diagnosis, monitoring, and treatment planning of neurological conditions.

### 3.4 Selection of brain segments to compare

For this study, we focused on comparing volumetric measurements of major brain structures, such as whole brain volume, cerebral white matter, and cerebellum; cerebral gray matter was not included since it occurs from subtracting white matter from the whole brain. Structures of hippocampus, thalamus, amygdala, and putamen were selected due to their clinical relevance and their association with neurodegenerative diseases of Alzheimer's disease, dementia, and multiple sclerosis (AbuHasan et al., 2020; Fogwe et al., 2020; Ghandili & Neuroanatomy, 2020; Torrico & Neuroanatomy, 2020). The ICV was also included, because it is used from both NQ and VB as normative data to express percentages of other brain structures, due to the fact that ICV remains constant in an adults’ life.

### 3.5 Statistics

The type of our data, NQ and VB measurements from the abovementioned brain segments, were paired, test–retest data, as they were performed on the same subjects (Ross et al., 2018).

For the comparison of the mean differences of the volume measurements from NQ and VB, the two‐tailed paired *t*‐test was used to test the null hypothesis that the average of the differences between the two software was zero, with a significance level a = .05 and 95% level of acceptance. If the calculated possibility (*p*‐value) is less than .05, it means that statistically the difference between the paired observations is significantly different from zero (Lee et al., 2021; Ross et al., 2018). The *t*‐value measures the size of difference relative to variations of the sample, the greater the *t*‐value, the greater the evidence against the null hypothesis.

For this study, the subjects were rated by the same ratters (NQ and VB software) with relevant systemic differences. The interclass correlation (ICC), model of the same ratters, and the type of absolute agreement were used to test the inter‐method reliability between NQ and VB. For interpretation of ICC value, ICC < .50, .50 < ICC < .75, .75 < ICC < .90, and .90 < ICC indicate poor, moderate, good, and excellent reliability, respectively (Koo & Li, 2016; Lee et al., 2021; Ochs et al., 2015; Yim et al., 2021).

Linear regression analysis was used to examine whether it is possible to transform data from one software to the other. The output has the form of a linear equation, which in this case takes the form: (VB) = a(NQ) + b where (VB) set to be the dependent variant and (NQ) the independent variant. The *R* squared coefficient determines the proportion of variance in the dependent variable that can be explained by the independent variable, *a* value closer to 1 means stronger linearity.

For the paired *t*‐test and the ICC statistical calculations, IBM SPSS Statistical Software, version 28.0.1.0 was used. For the graphical representation and regression of the square coefficient, Microsoft Excel software was used. The University of West Attika provided the user licenses for both software.

### 3.6 Manual measurement

If there is a major difference between the measurements of NQ and VB, and in order to state an established opinion on the subject, five patients (out of the total) were randomly selected for manual measurements to be performed on them. Axial and coronal plane images of 2 mm thickness were reformatted from the original 3D T1 sequence. An experienced neuroradiologist traced the anatomic boundaries of the segment on the images. The manual volume measurement was performed in two planes, and the average value of the measurement was considered, in order to minimize any errors in the procedure. The procedure was performed using Xinapse Systems, JIM8 version1.8.0_241.

## 4 RESULTS

For every brain segment and each volumetry software (NQ and VB), the mean values, percentage difference, mean differences, standard deviations, ICC, and *R* square, all appear in Table 1.

**TABLE 1:** The results of the measurements of each brain segment using NeuroQuant (NQ) and volBrain (VB) software and statistics are presented. Sample size is 56 (22Male/34Female).

	Average volume			*t*‐Test					
				Paired differences					
(1)	(2) NQ in cm^3^	(3) VB in cm^3^	(4) % Absolut difference	(5) Mean	(6) Std. deviation	(7) *t*‐Value	(8) *p*‐Value	(9) Intraclass correlation coefficient	(10) Linear regression *R* ^2^
Intracranial cavity	**1479.47**	**1359.18**	8.85	120.29	27.81	32.369	<.001	0.819	0.96
Whole brain	**1139.32**	**1118.43**	1.87	20.88	16.11	9.700	<.001	0.989	0.98
Cerebellum	**133.23**	**129.69**	2.73	3.41	2.95	8.664	<.01	0.980	0.97
Cerebral white matter	**439.40**	**408.04**	7.69	31.36	37.69	6.228	<.001	0.829	0.69
Amygdala	**3.29**	**1.48**	122.3	1.81	0.52	26.304	<.001	0.108	0.39
Hippocampus	**7.76**	**7.55**	2.78	0.22	0.74	2.207	<.032	0.818	0.59
Putamen	**10.49**	**7.57**	38.57	2.88	1.07	20.209	<.001	0.393	0.62
Thalamus	**13.83**	**9.71**	42.43	4.11	1.20	25.556	<.001	0.499	0.81

*Note*: For each selected brain segment (in rows), the average volume calculated from each software appears expressed in cubic centimeters (column 2, NQ, column 3, VB), followed by the percentage of their absolute difference (column 4). In columns 5, 6, 7, and 8, *t*‐test analysis is presented with mean difference, standard deviation, *t*‐value, and *p*‐value, respectively. Intraclass correlation coefficient presented in column 9. *R* square from linear regression analysis presented in the last column 10.

Analytical presentation of data for each segment (NQ average volume in cubic cm, VB average volume in cubic cm, absolute percentage of difference, paired *t*‐test *p*‐value, ICC, *R* square for linear regression): intracranial cavity (1479.47, 1359.18, 8.85%, < .001, .819, .96), whole brain (1139.32, 1118.43, 1.87%, < .001, .989, .98), cerebellum (133.23, 129.69, 2.73%, < .001, .980, .97), cerebral white matter (439.40, 408.04, 2.73%, < .001, .829, .69), amygdala (3.29, 1.48, 112.3%, < .001, .108, .39), hippocampus (7.76, 7.55, 2.78%, < .032, .818, .59), putamen (10.49, 7.57, 38.57%, < .001, .393, .62), and thalamus (13.83, 9.71, 42.43%, < .001, .499, .81).

**FIGURE 1:**
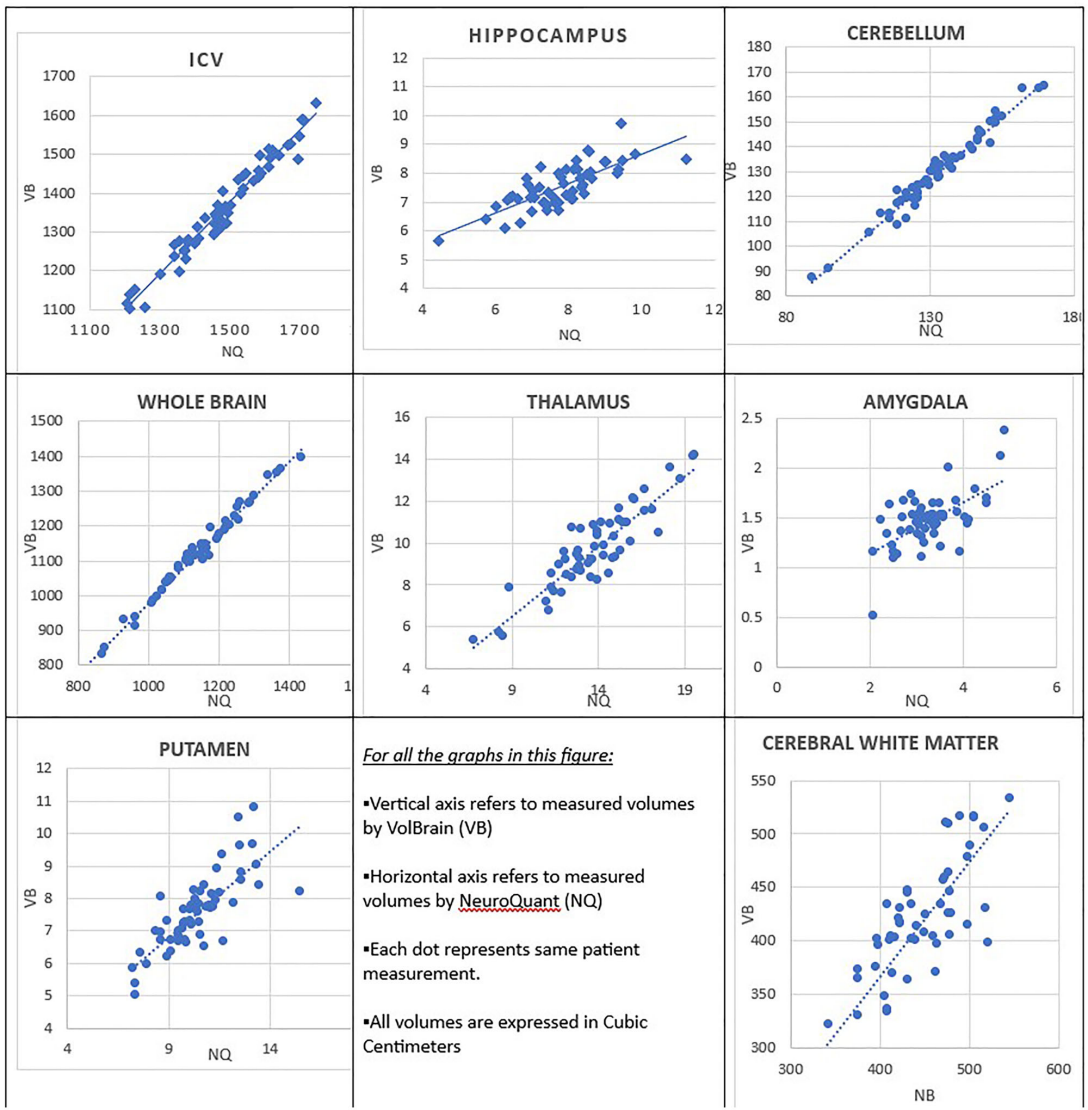
Graphical representation of the results. Each graph represents the volumetric measurements of a brain segment that appear on the graph title. On the vertical (*y*) axis appears the measurements of volBrain (VB), and on the horizontal (*x*) axis appears the measurements of NeuroQuant (NQ). All volumes are expressed in cubic centimeters.

In Figure 1, the diagrams for each brain segment are presented. Each dot represents a patient's measurements from VB (vertical axis) and NQ (horizontal axis), all expressed in cubic centimeters. The resulting equations from linear regression analysis by the means of (VB) = *a* (NQ) + *b* (in the form of *y* = *ax* + *b*) are as follows: intracranial cavity, *y* = .924*x* − 7.433; whole brain, *y* = 1.018*x* − 41.426; cerebellum:

*y* = 1*x* − 3.387; cerebral white matter, *y* = 1.078*x* − 65.708; amygdala, *y* = .262*x* + 0.613; hippocampus, *y* = .509*x* + 3.584; putamen, *y* = .529*x* + 2.043; thalamus, *y* = .669*x* + 0.459.

**FIGURE 2:**
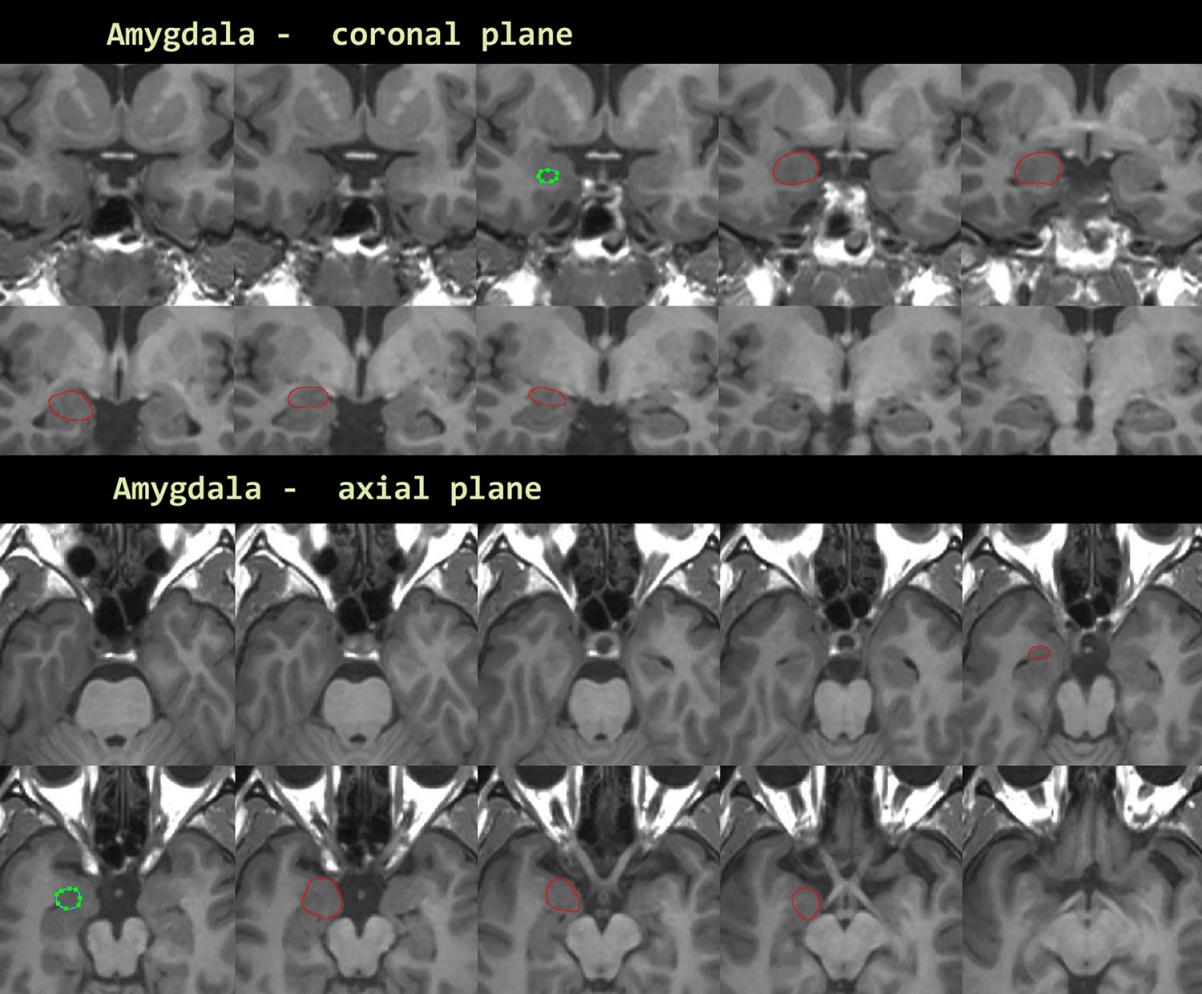
Snapshot from JIM8 screen that presents for one individual, the manual segmentation for right amygdala volume measurement in two planes coronal (above) and axial (below) under the instruction of an experienced neuro‐radiographer.

Amygdala is the brain segment where NQ and VB exhibited the greatest difference between them. Because the procedure is time consuming, manual volumetric measurements were performed only on the right amygdala. A snapshot of the mandatory measurement is presented on Figure 2. Manual volumetry of the right amygdala measured an average volume of 1.181 cm^3^. For the same sample, the automate calculated volume of the right amygdala was 1.716 cm^3^ for NQ and 0.786 cm^3^ for VB.

## 5 DISCUSSION AND CONCLUSION

When a measurement of a material body is performed, using different methods and techniques, the results are expected to vary due to procedural variations of each method. Nevertheless, these variations are not expected to be statistically significant.

All measurements made by NQ had bigger value than those made by VB for all segments. This finding agrees with other similar comparisons of NQ with FreeSurfer software (Ochs et al., 2015). The percentage of these differences was less than 5%, which is an acceptable error percentage (Keller & Roberts, 2009; Rehagen, 2021), in the segments of whole brain, cerebellum, and hippocampus; between 5% and 10%, in cerebral white matter and intracranial cavity; and well above 30% in putamen, thalamus, and amygdala. The paired *t*‐test analysis presents significant statistical differences (*p* < .001) in all segments but hippocampus (*p* = .032).

It would be useful if we had a “gold standard” for similar brain segments measurements, besides any other automated volumetry software, to compare and find out which software is closer to reality. Unfortunately, a search in the bibliography (Brabec et al., 2010; Kayaci et al., 2018; Keller et al., 2012; Kumar et al., 2014; Ochs et al., 2015) shows that produced measurements of brain segments were made with different methods and tools and, therefore, any comparison with our data would be unreliable.

For the extreme differences between measurements in the case of the amygdala, we cannot provide any explanation. Our manually performed calculations were also far different from both NQ and VB. Perhaps the boundaries of the anatomical region of amygdala are not well determined or are determined with more than one way.

For the areas of brain were all the segments with significant differences placed, a reasonable hypothesis is that tissue contrast in these segments' areas is poor enough for the software to accurately evaluate them. Reid et al.(2017) mentioned difficulty in segmenting thalamus stems due to T1 signal properties. Although T1 weighted MR sequence is considered to provide the best contrast for segmentation, a combined analysis from another sequences, like T2 FLAIR, may overcome this difficulty. Another study by Derix et al.(2014) suggested that scanning under ultrahigh field of 7.0T, with T1 weighted isotropic sequence, successfully overcomes the difficulty of distinction of the amygdalo‐hippocampal border of conventional scans. So, analysis from scans performed in a 7T scanner may have shown smaller differences, statistical not significant.

Also, taking into consideration that the accuracy in volume measurement of different structures is highly dependented on the definition of the anatomic structures in specific software, perhaps the initial hypothesis of absolute measurement was overestimated (Liu et al., 2020).

The comparison of NQ and VB software presented significant differences in the measurements of most of the brain segments we selected for this study. This indicates that the absolute values of the measurements cannot be considered reliable in all cases. The automated brain volume software proved not to be a satisfying quantitative tool.

The agreement of both software in the measurement of hippocampus means that for this and only segment, automated volumetry is a good quantitative tool. It could be quite safely used in clinical evaluation of pathological cases of dementia or epilepsy, which are strongly related to hippocampus atrophy.

ICC value shows poor reliability on amygdala, putamen, and thalamus; good reliability on intracranial volume, cerebral white matter, and hippocampus; and excellent reliability on whole brain and cerebellum.

In all but amygdala measurements, there was a good to excellent linear correlation. This means that it is possible to transform a volume measurement taken from one software as it could be measured from the other, using a simple linear equation of the form (VB) = *a*(NQ) + *b*.

The good to excellent linear regression and intraclass correlation coefficients, determined in most measurements, indicate that NQ and VB software packages can detect the relative size of each brain segment. Therefore, automated brain volumetry software can be a reliable qualitative tool for the treatment of patients with brain atrophy related diseases or other conditions related to changes of the volume of various brain segments. It is worth mentioning that other studies (Lee et al., 2021; Pareto et al., 2019; Yim et al., 2021) comparing other automated brain volume software like FreeSurfer and FIRST have reached to similar conclusions.

The case is not to decide which software is superior. The use of any automated brain volumetry software, at least between VQ and VB, is strongly recommended for research and clinical studies to evaluate and interpret a patient's condition (Stelmokas et al., 2017). But changing software, or scanning parameters, during a research project or a patient's monitoring should be avoided as misleading, because severe errors may occur.

## AUTHOR CONTRIBUTIONS

**Panagiotis Koussis**: Conceptualization; investigation; writing—original draft; data curation; writing—review and editing. **Panagiotis Toulas**: Visualization. **Demetrios Glotsos**: Project administration. **Eleni Lamprou**: Data curation; methodology. **Demetrios Kehagias**: Project administration; validation. **Eleftherios Lavdas**: Supervision.

## CONFLICT OF INTEREST STATEMENT

This research did not receive any specific grant from funding agencies in the public, commercial, or not‐for‐profit sectors. There are no potential financial conflicts of interest.

## FUNDING INFORMATION

Not applicable

### PEER REVIEW

The peer review history for this article is available at https://publons.com/publon/10.1002/brb3.3320

## Data Availability

My manuscript data will be sent as electronic supplementary material if needed.
